# First Birth of Cheetah Cubs from In Vitro Fertilization and Embryo Transfer

**DOI:** 10.3390/ani10101811

**Published:** 2020-10-05

**Authors:** Adrienne E. Crosier, Julie Lamy, Priya Bapodra, Suzi Rapp, Morgan Maly, Randy Junge, Holly Haefele, Jason Ahistus, Jenny Santiestevan, Pierre Comizzoli

**Affiliations:** 1 Smithsonian Conservation Biology Institute, National Zoological Park, Front Royal, VA 22630, USA; lamyj@si.edu (J.L.); Malym@si.edu (M.M.); santiestevanj@si.edu (J.S.); 2 Columbus Zoo and Aquarium, Powell, OH 43065, USA; Priya.Bapodra@columbuszoo.org (P.B.); Suzi.Rapp@columbuszoo.org (S.R.); Randy.Junge@columbuszoo.org (R.J.); 3 Department of Biological Sciences, North Carolina State University, Raleigh, NC 27695, USA; 4 Department of Molecular Biomedical Sciences, North Carolina State University, Raleigh, NC 27695, USA; 5 Fossil Rim Wildlife Center, Glen Rose, TX 76043, USA; hollyh@fossilrim.org (H.H.); jasona@fossilrim.org (J.A.); 6 Smithsonian Conservation Biology Institute, National Zoological Park, Washington, DC 20008, USA

**Keywords:** assisted breeding, cheetahs, ovum pick-up, in vitro fertilization, embryo transfer

## Abstract

**Simple Summary:**

Although the cheetah is the most studied of all wild felid species, reproduction of cheetahs in zoological settings has never been self-sustaining. A large proportion (~30%) of the Association of Zoos and Aquariums population is excluded from breeding due to advanced age, health, behavior, or management issues. Development of assisted reproductive technologies (ARTs) to enable the genetic contribution of sub-fertile individuals is now a priority research and management focus. We have previously demonstrated that aging females produce eggs that have the same developmental competence as young females. The objective of the present study was to produce embryos in vitro from older donor oocytes and transfer them into younger recipients to obtain pregnancies and live births. Good quality oocytes were collected from three synchronized donors, fertilized in vitro with frozen-thawed semen, and cultured for two days. Resulting embryos were transferred into the oviduct of three synchronized recipients. Pregnancies were monitored via fecal levels of progestogens, ultrasonography, and radiography. Two cubs were born naturally after 90 days of gestation, representing the first cheetah births resulting from the transfer of embryos produced in vitro.

**Abstract:**

Approximately 30% of the Association of Zoos and Aquariums cheetah population (~350 total animals) is unlikely to breed naturally due to advanced age, health, or behavioral issues. Aging cheetah females (≥9 y old) are unlikely to become pregnant via natural breeding if they are nulliparous. We previously demonstrated that oocytes recovered from aged females were of similar quality compared with those recovered from younger females (2–8 y old). We hypothesize that transfer of 4–8 cell embryos produced by in vitro fertilization with oocytes from old donors could result in pregnancy after transfer into younger recipients. Female cheetahs (*n* = 3 aging donors and *n* = 3 young recipients) received 300 IU equine Chorionic Gonadotropin (eCG) and 3000 IU Luteinizing Hormone (LH) while fecal metabolites of estrogens and progestogens were closely monitored. At 28 h post-LH injection, oocytes were aspirated laparoscopically from donors and inseminated in vitro with cryopreserved sperm. After 48 h of in vitro culture, resulting embryos (4–8 cells) were transferred into the oviducts of recipient females. Pregnancy was confirmed in one recipient via ultrasound 32 days after transfer and by radiograph 62 days after transfer. Two cubs were born naturally after 90 days of gestation, representing the first cheetah births resulting from transfer of embryos produced in vitro.

## 1. Introduction

Cheetahs are highly charismatic and popular exhibit animals with ~25% of facilities in the Association of Zoos and Aquariums (AZA) exhibiting the species [1]. The wild cheetah population is decreasing in numbers due to habitat loss and fragmentation, loss of prey base, and poaching, and there are only ~7100 cheetahs remaining in the wild, occupying less than 10% of their historic range [2]. The AZA cheetah population is managed through a Species Survival Plan (SSP), and currently, there are ~350 cheetahs in this managed population, representing a genetic reservoir for their free-ranging counterparts. Effective breeding population size (Effective population size relative to the entire population) in the AZA has improved over the last decade (from 0.15 to 0.25), which is higher than the global ex situ average for this species (0.16); however, it is still below the target of 0.35 for this population [1]. Reproductive success within the SSP is poor, with less than 20% of females producing offspring.

A challenge to cheetah management is the removal of 30% of the population from the breeding matrix due to health, advanced age, behavior, and/or fulltime participation as education animals. The ability of nulliparous female cheetahs to reproduce after the age of nine is drastically decreased as females develop significant and severe uterine pathologies as young as age six if they have not produced a litter [3]. However, oocytes from these aged females are viable, with high success rates in fertilization in vitro as well as early embryonic development [3]. We also have shown that cheetah embryo production in vitro is successful using cryopreserved sperm cells [3]. The ability to use frozen-thawed sperm for embryo production allows the transfer of gametes from males to numerous institutions without transport of live animals. The harvesting of genetic material from aged, non-breeding females would enable the reproduction of cheetahs and ensure their genes are incorporated into the population. The ability to reproduce cheetahs that are otherwise unable or unwilling to breed on their own is a critical step to improving genetic and population management of this species. The objective of the study was to recover oocytes from aged and non-breeding females, along with the harvesting of sperm followed by cryopreservation, for production of embryos via in vitro fertilization (IVF) and transfer into younger recipient female cheetahs.

## 2. Materials and Methods

### 2.1. Animals and Husbandry

All animal-related procedures were approved by the National Zoological Park’s Institutional Animal Care and Use Committee (IACUC) and similar committees at each of the partner institutions. Semen was collected from adult male cheetahs at the Fossil Rim Wildlife Center (FRWC, *n* = 1, ~3 y of age) and at the Smithsonian Conservation Biology Institute (SCBI, *n* = 1). The male at SCBI (#9459) contributed two samples, with approximately a 15-month interval between collections, and was ~2 y old at the first collection and ~3 y old at the second. This male was housed with his brother in an outdoor enclosure (200′ × 85′) with an outdoor, unheated shelter and indoor access to heat. This male was fed a commercially produced beef diet (Central Nebraska Packing Inc., North Platte, NE, USA) with weekly supplements of bones; water was provided ad libitum. The male at the FRWC contributed a single semen sample and was housed with his brother in an outdoor enclosure (250′ × 100′). This male was fed a commercially produced horsemeat diet (Milliken Meat Products Ltd., Markham, ON, Canada), with weekly supplements of bones, and water was provided ad libitum. The single donor female (Donor 1, 7 y of age) and the single recipient female (Recipient 1, 6 y of age) at SCBI were housed individually in outdoor enclosures (125′ × 150′) with an outdoor, unheated shelter and indoor access to heat. These females were fed a commercially produced beef diet (Central Nebraska Packing, Inc.) with weekly supplements of bones. Water was provided ad libitum. The two donor females (Donor 2 and Donor 3, 9 and 6 y of age, respectively) and the two recipient females (Recipients 2 and 3, both 3 y of age) at the Columbus Zoo (CZ) were housed in an enclosure with indoor and outdoor access (24′ × 24′ total) with regular access (5–7 days per week) to a large exhibit and exercise yard. At CZ, cheetahs were fed a commercially produced horsemeat diet (Central Nebraska Packing, Inc.) and water was provided ad libitum. Expect for Recipient 1, none of the females involved in that study had a litter before.

### 2.2. Semen Collections

Semen was collected via electroejaculation under a surgical plane of anesthesia using previously established protocols [4,5]. The male cheetahs were anesthetized with a combination of midazolam, ketamine, and medetomidine at SCBI and a combination of midazolam, butorphanol, ketamine, and medetomidine at Fossil Rim. During the anesthesia events, each male was monitored for appropriate respiration, heart rate, and temperature. Testes were measured with calipers. Semen was collected by insertion of a 1.9 cm rectal probe followed by a total of 80 stimulations that were conducted in three series with 30 simulations in the first one, 30 stimulations in the second one, and 20 stimulations in the last one. Samples were assessed for volume and pH, and an aliquot (5–10 µl) of raw semen was fixed in 0.3% glutaraldehyde in Phosphate Buffered Saline (PBS) and assessed for structural morphology as previously described [4,5]. Samples were resuspended 1:1 in Ham’s F10 culture medium (HF10, Irvine Scientific, Santa Ana, CA, USA) supplemented with 20 mM HEPES, 5% (v:v) fetal calf serum (Irvine Scientific) and 1 mM pyruvate, 2 mM glutamine, 10,000 IU/mL penicillin, 100 µg/mL streptomycin and 20 mg/mL neomycin (Millipore Sigma, Rockville, MD, USA), and percent motility and forward status (0–5) were assessed using standard criteria [4]. Samples were combined and concentration determined using a hemocytometer. Sperm cells were concentrated by centrifugation (100 × g for 8 min) and the supernatant was discarded. Resulting pellets were resuspended in Test yolk-buffer medium with 0% glycerol (refrigeration medium, Irvine Scientific). Samples were cooled in a water bath to 4 °C for ~2.5 h. An equal volume of Test yolk-buffer with 8% glycerol was added incrementally (¼ volume added, 15 min later ¼ volume added and then 15 min later the final ½ volume added) over a period of 30 min. Sterile 0.25-mL straws were loaded with ~100 µL sperm suspension at a concentration of ~60 × 10^6^ motile cells/mL. Straws were frozen in liquid nitrogen in two steps: straws were placed 3 inches above the liquid nitrogen for 1 min, then 1 inch for 1 min, and then immersed into liquid nitrogen. Straws were stored in liquid nitrogen at SCBI until the IVF.

### 2.3. Hormonal Stimulations and Laparoscopic Oocyte Aspiration

Oocytes were harvested from donor females via laparoscopy using previously described methods [3]. Each donor female received 300 IU equine Chorionic Gonadotropin (eCG) (i.m.) followed ~86 h later by 3000 IU Luteininzing Hormone (LH) (i.m.). Approximately 28 h following LH injection, females were anesthetized with a combination of midazolam, butorphanol, and medetomidine. Anesthesia was maintained with isoflurane and the animals were prepared for laparoscopy. For laparoscopy, a 2-mm diameter Verres probe was inserted transabdominally and the abdomen was insufflated with filtered air using a hand pump. A 5-mm diameter laparoscope (Olympus Co, Center Valley, PA, USA) was passed through a trocar cannula inserted in a ~3 mm incision along the abdominal midline to visualize the reproductive tract. Number of all ovarian structures (follicles over 2-mm diameter) were recorded. A secondary trocar cannula (5 mm) was inserted through a small (~3 mm) incision made along the midline to allow insertion of forceps to stabilize the ovary for oocyte aspiration. For oocyte recovery, a 22-ga sterile spinal needle attached to PE100 polyethylene tubing and aspiration device were utilized. This needle was inserted through the abdominal wall and into the ovarian follicle for extraction of oocytes directly from preovulatory follicles. Oocytes were collected in a sterile vial containing Hams medium with HEPES and supplemented with heparin [3].

### 2.4. Semen Preparation and In Vitro Fertilization

Recovered oocytes were graded according to standard criteria for felids [3]. Briefly, Grade 1 (‘excellent’) oocytes had a uniformly dark cytoplasm and multiple layers of expanded cumulus cells. Grade 2 (‘good’) oocytes had a similar cytoplasmic appearance, but with fewer layers of expanded cumulus cells. Grade 3 (‘average’) oocytes had a slightly vacuolated and non-uniform cytoplasm as well as few (1–3) layers of cumulus cells. Grade 4 (‘poor’) quality oocytes were characterized by vacuolated and inconsistent cytoplasm as well as a lack of cumulus cell investment. Only Grade 1 and 2 oocytes were retained for fertilization.

Selected oocytes were washed twice in small petri dishes containing 3 mL of SAGE medium (Quinn’s Advantage Protein Plus Blastocyst medium, SAGE medium, Trumbull, CT, USA). Oocytes were placed in 45 µL droplets of SAGE medium overlaid with 700 µL of mineral oil (Sigma, Rockville, MD, USA) in a 4-well petri dish (Nunc, Thermofischer, Waltham, MA, USA) with ~8–10 oocytes per well. Oocytes were then inseminated in vitro with cryopreserved sperm. Individual straws were thawed for 10 s in air followed by 30 s in a 37 °C water bath. Straw contents were dispensed into a sterile Eppendorf tube and assessed for motility. Each sperm suspension was overlaid with 50 µL fresh Hams medium and cells were allowed to swim-up into the medium for 30 min at 37 °C in a dry incubator. Sperm concentration was checked and 5 µL of sperm suspension were added in each droplet containing selected oocytes. Final sperm concentration for IVF was about 1 million motile sperm/mL. Fertilization petri dishes were then placed at 38.5 °C in a humidified atmosphere with 5% CO_2_ in a MINC bench-top incubator (Cook Medical, Bloomington, IN, USA) for 18–22 h.

### 2.5. Embryo Culture and Laparoscopic Embryo Transfer

After fertilization, oocytes were placed in a small petri dish containing 3 mL of SAGE medium and stripped from surrounding cumulus cells and dead sperm cells by pipetting with a fine mouth pipette. Presumptive zygotes were then washed in 500 µL of SAGE medium and placed in 50 µL droplets of SAGE medium overlaid with 700 µl of mineral oil (~10 embryos/well) and allowed to develop in a humidified atmosphere with 5% CO_2_ for 24 h.

Only cleaved embryos were transferred into recipients via laparoscopy using previously described methods [3]. Each recipient female received 300 IU eCG (i.m.) followed ~86 h later by 3000 IU LH (i.m.). At approximately 52 h after LH injection, females were anesthetized with a combination of midazolam, butorphanol, and medetomidine. Anesthesia was maintained with isoflurane and intra-oviductal embryo transfer was performed as follows. Number of all ovarian structures (follicles over 2-mm diameter) and ovulation sites were recorded. After insertion of the same instruments mentioned for the oocyte aspiration (see above in Section 2.3), the ovarian bursa was extended and everted with the grasper to allow visualization of the abdominal oviductal ostium. An intravenous catheter (18G, 32 mm length; Terumo Medical Corporation, Elkton, MD, USA) was inserted percutaneously proximal to the ovarian bursa, and 23ga PE50 tubing was passed through a modified 22G needle (i.e., a blunted stylet from a 20G IV catheter, 68 mm length; Terumo Medical Corporation), all of which was inserted through the catheter approximately 1 cm deep into the oviductal ostium. A 1-mL syringe was affixed to the tubing to apply gentle pressure to dispense the embryos into the ostium. A total of 20 embryos were transferred to the three recipients. Each recipient was paired with a donor female (see Section 3). Due to the large number of transferrable embryos from Donor 3, embryos were transferred into two recipients (2 and 3).

### 2.6. Pregnancy Monitoring Through Endocrinology and Ultrasonography

Fecal samples (4–5 per week) were collected from donor and recipient females for 9 total weeks. Individual samples were placed into clean, labeled plastic bags and stored frozen (−20 °C) until freeze-dried (Lyophilizer; Labconco, Kansas City, MO, USA). Enzyme immunoassays (EIAs) for measuring estradiol and progestogens have been previously described and validated in our laboratory at SCBI for cheetah feces [3,6]. In brief, the EIAs were performed in 96-well microtiter plates (Nunc-Immuno, Maxisorp Surface; Fisher Scientific, St. Louis, MO, USA) with assay-specific standards (Steroloids, Inc., Newport, RI, USA) and diluted fecal extracts assayed in duplicate. Baselines for estradiol (before receiving eCG) and for progestogens (following LH injection) were calculated for each cheetah using an iterative process that excluded values exceeding the mean + 1.5 Standard Deviation [6]. At the Columbus Zoo, female cheetahs were evaluated for pregnancy via voluntary ultrasonography (Sonosite Edge II machine, with the iC60 5-2 MHz Transducer, Bothell, WA, USA). Pregnancy was detected in one recipient 32 days after embryo transfer (34 days after IVF/conception). Pregnancy was reconfirmed and cub number determined in this same recipient on 62 days post-transfer (64 days post-IVF) by radiography.

### 2.7. Genetic Testing

Whole blood samples were collected from all donor and recipient females as well as the semen donor males during the above-described procedures. Whole blood was collected from the two cubs during routine veterinary exams shortly after birth. All blood samples were preserved in EDTA and stored at −80 °C. DNA was extracted using a commercial kit (DNeasy Blood and Tissue Kit, Qiagen, Germantown, MD, USA) and stored at −20 °C. Samples were genotyped with a panel of nine cheetah-specific microsatellite markers [7]. Fragments were separated with a 3730 xl DNA Analyzer (Applied Biosystems, Foster City, CA, USA). Genotypes were assessed using Geneious v 10.2.3. All individuals (*n* = 6) were genotyped at all nine loci. Parentage was assigned using CERVUS v. 3.0.7 [8] to calculate the natural log of the overall likelihood ratio (LOD score) for each candidate parent and for each offspring-dam-sire trio. Candidate parents for each offspring included two dams, the egg donor (Donor 3) and the recipient female (Recipient 2), and two sires, the IVF sperm donor and an unrelated male. The simulation confidence interval parameters were 95% at the strict level and 80% for relaxed.

## 3. Results

### 3.1. Testicular Biometry and Semen Characteristics

Males used for the study had similar testicular sizes and produced semen samples of comparable quality (Table 1). pH measurements for the three samples were similar to previous reports in cheetahs (8.7, 9.0 and 8.7 for Samples 1, 2, and 3, respectively) [4,5]

### 3.2. Donor Response to the Stimulation and Oocyte Collection

**Figure 1:**
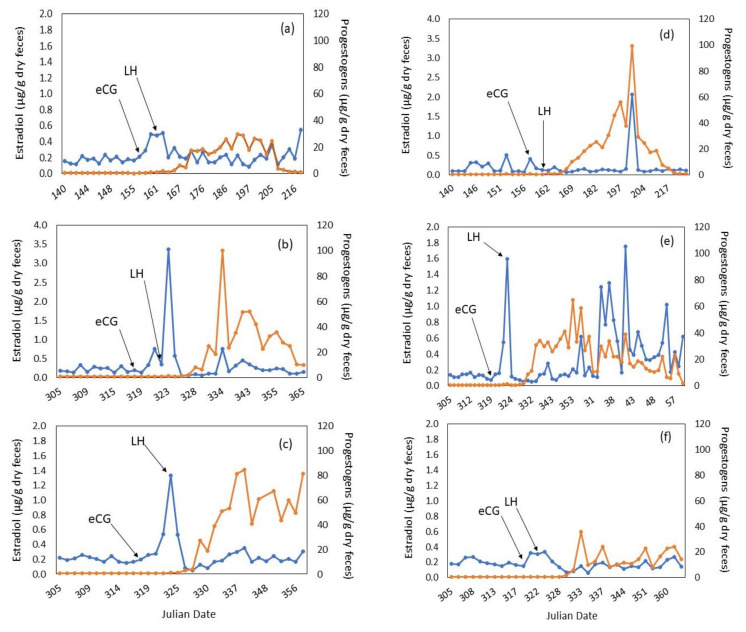
Fecal hormone concentrations for donor (**a**–**c**) and recipient (**d**–**f**) cheetah females. Estradiol (blue line) and progestogen (orange line) metabolites are depicted for three oocyte donor cheetahs: (**a**) Donor 1, 7 y old donor at SCBI; (**b**) Donor 2, 9 y old donor at Columbus Zoo; (**c**) Donor 3, 6 y old donor at Columbus Zoo, and three recipient cheetahs: (**d**) Recipient 1, 6 y old recipient at SCBI; (**e**) Recipient 2, 3 y old recipient at Columbus Zoo and (**f**) Recipient 3, 3 y old recipient at Columbus Zoo. Day of administration of each equine Chorionic Gonadotropin (eCG) and Luteinizing Hormone (LH) are noted for each female. Note the differences in estradiol scale for (**b**,**d**).

**Figure 2:**
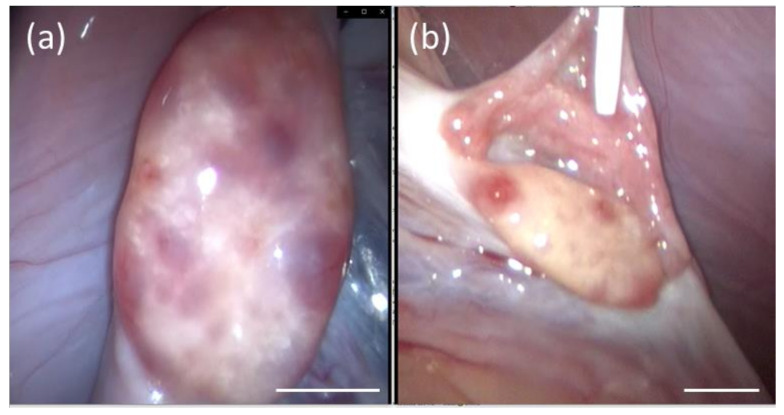
Representative pictures from laparoscopy. Ovary from Donor 3 (**a**) with preovulatory follicles (darker areas) that were then aspirated to recover the oocytes. Ovary with fresh ovulation sites (bright red spots) and oviduct from Recipient 2 (**b**) during oviductal embryo transfer. Bar = 10 mm.

The estradiol baseline concentrations for the three donor females before eCG dosing were: 0.15 µg/g dry feces, 0.15 µg/g dry feces, and 0.19 µg/g dry feces for Donors 1, 2, and 3, respectively. The first donor female (Donor 1; Figure 1a) had a poor hormonal response to the eCG and LH injections. The second donor (Donor 2; Figure 1b) had a delayed estradiol increase after LH injection. The third donor (Donor 3; Figure 1c) had an ideal response to eCG/LH stimulation with the estradiol peak occurring on the same day as the LH injection. Four to 6 days after oocyte aspiration (performed between 28–28.5 h post-LH injection), all donors had elevated progestogen concentrations (elevation is defined as 3× baseline for 5 consecutive days). The three donors had the following baseline progestogen concentrations for the first 6 weeks post-LH injections: Donor 1, with embryos not producing cubs, 15.3 µg/g dry feces (Figure 1a); Donor 2, with embryos not producing cubs, 12.9 µg/g dry feces (Figure 1b) and Donor 3, with embryos producing cubs, 45.2 µg/g dry feces (Figure 1c). The three recipients had the following baseline progestogen concentrations for the first 6 weeks post-LH injections: Recipient 1, did not produce cubs, 15.6 µg/g dry feces (Figure 1d); Recipient 2, produced live cubs, 21.1 µg/g dry feces (Figure 1e) and Recipient 3, did not produce cubs, 7.8 µg/g dry feces (Figure 1f). During laparoscopy, it was confirmed that all donor females developed follicles in response to the eCG/LH stimulation (Table 2, Figure 2a). No donor females had corpora hemorrhagica (CH) or corpora lutea (CL) on their ovaries (Figure 2a) at laparoscopy. Oocytes collected were classified based on quality grade as described above. Only oocytes with quality Grade 1 and 2 were inseminated (Table 2).

### 3.3. Embryo Production and Transfer

After thawing, the 3 semen samples used for in vitro fertilization (IVF) had good percentages of sperm motility (Table 3). An average concentration of 0.92 ± 0.27 million total motile sperm /mL (ranging from 0.36 to 1.2 total motile sperm/mL) was added to each drop containing oocytes. Following IVF, number and proportions of cleaved embryos on Day 1 and Day 2 were similar between donors (Table 4). Embryos (2-cell or 4-cell stages) were then isolated before the embryo transfer (Figure 2b).

The estradiol baseline concentrations for the three recipients before eCG dosing were 0.09 µg/g dry feces, 0.12 µg/g dry feces and 0.17 µg/g dry feces, for Recipients 1, 2, and 3, respectively. On the day of laparoscopy, the first recipient (Figure 1d) had not ovulated at the time of embryo transfer. An additional LH injection did result in ovulation according to the hormonal profile (Figure 1d). The second recipient (Figure 1e) had a perfect response to eCG/LH stimulation (estradiol peak on the same day as the LH injection) and was the female that established pregnancy. The third recipient (Figure 1f) had a poor hormonal response to the eCG/LH stimulation. Recipients 2 and 3 had CH/CL present at the time of embryo transfer (Table 2). The recipient with the most CL (*n* = 9) was the one that established pregnancy. All recipients received embryos (Table 4). The recipient that established pregnancy received the most embryos (*n* = 9; Table 4).

### 3.4. Pregnancy Monitoring and Genetic Testing

**Figure 3:**
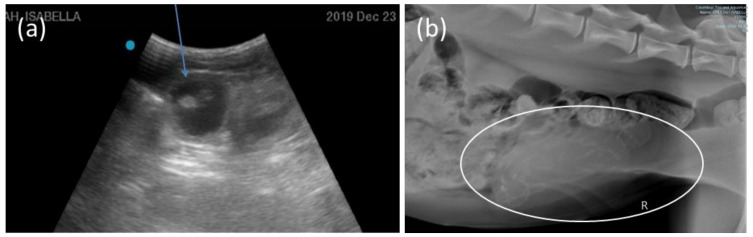
Pregnancy monitoring of recipient female by ultrasonography 32 days post-transfer, the arrow is pointing at the embryonic vesicle filled with fluid (**a**) and radiography 62 days post-transfer, mineralized skeletons of both cubs are visible within the circle (**b**).

**Figure 4:**
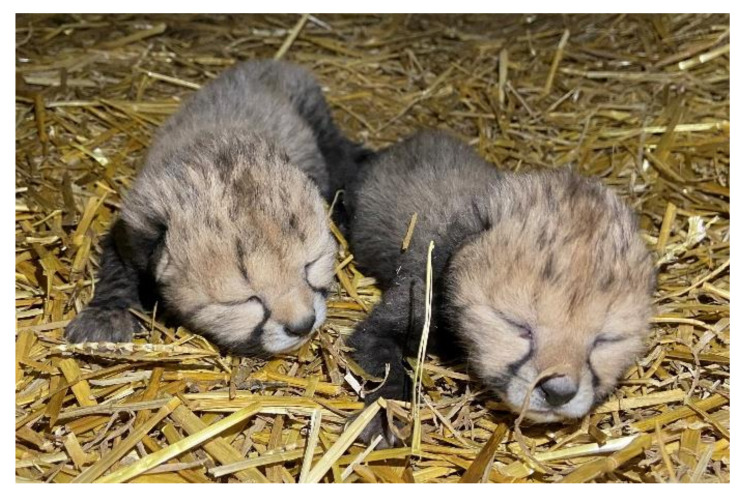
Cheetah cubs 2 days after birth.

All recipients had elevated progestogen concentrations 4–6 days after embryo transfer (Figure 1). The 3 recipients had the following average progestogen concentrations for the first 6 weeks post-LH injections: Recipient 1, 19.7 µg/g dry feces (Figure 1d); Recipient 2, female pregnant 25.5 µg/g dry feces (Figure 1e); Recipient 3 not pregnant 12.8 µg/g dry feces (Figure 1f). The pregnant female had the greatest increase during the first trimester (Figure 1e). Monitoring by ultrasonography and radiography confirmed the pregnancy (Figure 3a,b). The female gave birth to two cubs (one male and one female) 92 days following IVF of donor oocytes (90 days following transfer of 4–8 cell embryos). Cub weights on day 1 of life were approximately 480 g for the male and 350 g for the female cub (Figure 4).

Parentage was successfully resolved for both offspring. The IVF sperm donor and egg donor 3 were the most likely parental candidates for cub 1 and cub 2 with zero mismatching loci and trio LOD scores of 4.59 and 3.86, respectively. All other parental trio combinations contained genetic mismatches and negative LOD scores (Table 5).

## 4. Discussion

This is the first report of a birth of healthy cheetah cubs produced by IVF and embryo transfer. The cheetah population under human care serves as education ambassadors, as well as genetic and animal resources for their wild counterparts. The majority of globally managed cheetah populations experience poor reproductive success, with many animals removed from breeding for health and/or behavior reasons. Assisted reproduction in wild felids is challenging and few successes have been obtained to date.

The preparation and hormonal monitoring of the donor cheetahs in this study revealed differences between individuals that are consistent with previous studies from our laboratory [3,6]. The hormonal profile of the donor female having the best response to the hormonal stimulation (estradiol peak following eCG and LH injection at the apex of this peak) and producing oocytes leading to healthy cubs was different from the other two donors. This indicates that oocytes from that female may have had the best developmental competence. From previous studies, we know that cheetah females receiving eCG more than three days after a natural estradiol surge generate a significant estradiol peak following eCG, and produce higher quality oocytes [3,6]. The ovarian response of the recipient females after the hormonal stimulation also varied among individuals. Two of the recipient females had overall poor responses to the hormonal stimulation, resulting in low production of progestogens. The female that established pregnancy demonstrated an ideal response to the exogenous hormones.

The number and quality of oocytes collected from each female was consistent with previous results in cheetahs [3]. Similarly, quality of freshly collected semen samples, as well as post-thaw metrics of samples used for IVF, were comparable to our previous publications [3,4]. However, IVF conditions were slightly different in the present study as we used a new culture medium and a new type of incubator. Regardless, percentages of cleaved embryos were comparable with our previous studies [3]. In many domestic livestock species, late-stage embryos are transferred to recipient females. We chose to reduce the time of embryo culture, and transfer embryos early in development, to increase the chances of pregnancy establishment.

Additionally, our ability to transfer embryos directly into the oviduct of felids enables us to transfer early-stage, viable embryos. The transfer of early embryos into the oviduct is an approach that was developed in the domestic cat and that also proved to be successful in small wild felids [9]. Implementing assisted reproductive technologies in large cat species has always been more challenging for physiological reasons. This present success in the cheetah, therefore, is a considerable advance in our knowledge about cheetah physiology and overall benefit to large cat conservation. A previous and comparable success was obtained in tiger more than 30 years ago [10], which shows how difficult it is to make progress in those species.

Early pregnancy detection is a management challenge in large cats. Cheetahs are induced ovulators, demonstrating an increase in progestogens following mating [11]. However, a high proportion (~60%) of cheetah mating in the AZA population do not result in offspring production [12], making the diagnosis of actual pregnancy impossible with traditional steroid hormone analysis until after ~55 days post-mating [11,12]. Recent research on novel protein production, analyzed in cheetah fecal samples, has yielded promising results for future establishment of protocols for identifying pregnancy shortly after breeding events [12,13]. Other options for pregnancy diagnosis include ultrasonography and radiography. As with traditional steroid analysis, radiographs also are not accurate until after day 54 post-mating, when fetal bone mineralization has occurred [14]. Ultrasonography requires a tractable, trained female, and there are few opportunities for this in the managed cheetah population. Fortunately, the cheetah females at the CZ are all well-trained, allowing unique opportunities for non-sedated ultrasonography determination of pregnancy shortly after embryo transfer. A final, significant accomplishment from this research is the production of healthy cubs of normal size and normal growth.

## 5. Conclusions

More procedures are warranted, but the lessons learned from this trial will pave the way for integrating IVF and embryo transfer into the management of the cheetah population.

## Figures and Tables

**Table 1:** Testicular and raw semen metrics of male cheetahs.

Semen Sample	Testicular Volume (cm^3^)	Raw Semen Metrics			
		Volume (mL)	Concentration (×10^6^/mL)	Motile Sperm (%)	Normal Sperm (%)
Sample 1 (male 9659)	8.72	1.62	85.5	80	33
Sample 2 (male 9659)	10.60	1.10	29.5	60	31
Sample 3 (male 9613)	9.70	1.02	150.0	70	40

**Table 2:** Ovarian and oocyte metrics from donor and recipient cheetah females.

Donor and Recipient Females	Number of Ovarian Follicles/Female	Number of Ovulation Sites/Female	Number of Oocytes/Female *
Donor 1	18	None	18
Donor 2	8	None	12
Donor 3	20	None	18
			
Recipient 1	30	None	N/A ^1^
Recipient 2	5	9	N/A
Recipient 3	11	3	N/A

* Represents all oocytes of quality grades 1–2. N/A ^1^ indicates data are not applicable for this group of females.

**Table 3:** Sperm metrics post-thawing and after swim-up processing.

Semen Sample	Concentration (x 10^6^/mL)	Motile Sperm (%)	Total Sperm/IVF Drop	Donor Female
Sample 1 (male 9659)	13.0	50	3.9 × 10^4^	Donor 1
Sample 2 (male 9659)	12.5	80	5.0 × 10^4^	Donor 2
Sample 3 (male 9613)	14.0	80	5.6 × 10^4^	Donor 3

**Table 4:** Proportion of cleaved embryos obtained from each donor and number of embryos transferred to each recipient.

Donor Females	Number of Cleaved Embryos (and %) on Day 1 Post-Insemination	Number of Cleaved Embryos (and %) on Day 2 Post-Insemination	Number of Transferred Embryos	Recipient Females
Donor 1	5 (27.8)	5 (27.8)	5	Recipient 1
Donor 2	3 (25.0)	5 (41.7)	5	Recipient 3
Donor 3	5 (27.8)	10 (55.6)	9 and 1	Recipient 2 and 3

**Table 5:** Overall likelihood ratio (LOD score) for each candidate parent and for each offspring-dam-sire trio.

Offspring	Dam	Sire	Trio Typed Loci	Trio Loci Mismatches	Trio LOD Score
Cub 1	Donor 3	Sperm donor	9	0	4.59
	Recipient 2	Sperm donor	9	2	−4.79
	Donor 3	Unrelated male	9	4	−13.6
	Recipient 2	Unrelated male	9	6	−23.9
Cub 2	Donor 3	Sperm donor	9	0	3.86
	Recipient 2	Sperm donor	9	3	−9.84
	Donor 3	Unrelated male	9	5	−20.6
	Recipient 2	Unrelated male	9	6	−22.6
